# A Checklist Approach for Enhanced Outpatient Guideline-Directed Management in the Secondary Prevention of Atherosclerotic Cardiovascular Disease

**DOI:** 10.14797/mdcvj.907

**Published:** 2021-09-24

**Authors:** Mohamad B. Taha, Eleonora Avenatti, Daniel S. Li, Tirhas Ohonba, Miguel Cainzos-Achirica, Kershaw V Patel, Khurram Nasir

**Affiliations:** 1 Houston Methodist DeBakey Heart & Vascular Center, Houston Methodist Hospital, Houston, Texas, US; 2 Houston Methodist Hospital, Houston, Texas, US; 3 Center for Outcomes Research, Houston Methodist, Houston, Texas, US; 4 Center for Cardiovascular Computational & Precision Health, Houston Methodist, Houston, Texas, US

**Keywords:** atherosclerotic cardiovascular disease, secondary prevention, checklist, guidelines, evidence-based medicine

## Abstract

Compelling results from clinical trials supporting intensive risk-reduction therapies to reduce associated morbidity and mortality in patients with established atherosclerotic cardiovascular disease (ASCVD) provided the impetus for medical societies to integrate these evidence-based results into clinical practice guidelines. Current evidence, however, points toward gaps in the management of patients with established ASCVD. Some of these gaps are related to barriers to guideline implementation, and strategies are needed to overcome these barriers. In this review, we propose a framework incorporating comprehensive tools for enhanced guideline-directed management in secondary prevention of ASCVD. This aid includes a 13-point checklist with supporting educational and system-based tools for effective evidence-based pharmacological and nonpharmacological care. This proposed tool targets primary care providers and cardiologists in the outpatient setting who provide direct medical care for patients with established ASCVD.

## Introduction

Atherosclerotic cardiovascular disease (ASCVD), which includes coronary heart disease (CHD), cerebrovascular disease, peripheral artery disease (PAD), and aortic atherosclerotic disease, remains the leading cause of morbidity and mortality in the United States (US). Approximately 32 million US adults are living with established ASCVD.^1^ Those with a prior history of ASCVD are at increased risk for recurrent events, morbidity, and mortality.^2,3^

A goal of ASCVD secondary prevention is to delay or prevent downstream sequelae of a clinical event. To that end, several randomized clinical trials have demonstrated cardiovascular benefits of lifestyle interventions,^4,5,6,7^ treatment with high-intensity statin therapy or maximally tolerated statin therapy to achieve goal low-density lipoprotein cholesterol (LDL-C) levels,^8,9^ and management of diabetes, hypertension, dyslipidemia, and excessive weight among patients with a history of ASCVD.^10,11,12,13^ This was translated by medical societies to clinical practice guidelines with the goal of preventing recurrent ASCVD events and reducing associated morbidity and mortality. These guidelines are updated regularly to include the most recent therapeutic developments.

Despite high-quality evidence and the availability of formal guidance from cardiovascular societies, studies demonstrate that there are significant gaps in secondary prevention management of patients with established ASCVD. For instance, even though LDL-C lowering with statins is one of the most effective treatment strategies to reduce ASCVD morbidity and mortality, the use of all classes of lipid-lowering therapies has not matched guideline recommendations.^14,15,16,17,18^ Missed opportunities for initiation and intensification of secondary prevention treatments are likely related to several factors, including physician clinical inertia in managing treatment or failure to stay updated on guidelines, lack of experience with emerging novel therapeutics, cost obstacles, administrative burden in prescribing newer medications, and suboptimal patient adherence.

Checklists are organized tools implemented in workflows to guide users through accurate task completion, ensuring that simple standards are applied consistently. High-risk environments, such as the aviation industry, use checklists to avoid human error and prevent dangerous mistakes with an overall goal of improving outcomes. The checklist approach has been translated into several fields of medicine, including surgery, critical care, and emergency services.^19,20,21^ Recently, the American Heart Association (AHA) has developed a checklist for the prevention of recurrent strokes.^22^ However, there is not a well-established checklist for secondary prevention of overall ASCVD.

Barriers to guideline implementation include provider-, guideline-, and system-related factors. Providers may not be fully aware of the specific guidelines and current data supporting the recommendations or may disagree with them, and others may lack motivation to stay updated. Patients with established ASCVD who are cared for by cardiologists are more likely to receive guideline-recommended therapy than those only seen by primary care physicians,^23^ yet primary care physicians are oftentimes tasked with managing these patients. Therefore, readily available supportive tools might reduce observed gaps and barriers. Guideline-related barriers include their complexity, layout, and tendency to focus on patients with single disease entities. System-related factors include lack of resources and collaboration, time restrictions, and workload. Checklists and supporting tools can be used as a platform to disseminate guidelines and implement decision support systems, thereby potentially overcoming some of these barriers.^24,25,26^

This review provides a potential framework for developing a comprehensive tool consisting of a checklist and supporting materials to improve adherence to guideline-directed management in the secondary prevention of ASCVD. This tool is expected to be implemented by providers who manage patients with ASCVD in an outpatient setting.

## Checklist Development

### Patient Selection

The target patient population for the checklist and supporting tools includes adult patients with established ASCVD. A prior history of ASCVD is defined by the presence of any of the following: acute coronary syndrome, history of myocardial infarction, stable or unstable angina, coronary or other arterial revascularization, stroke, transient ischemic attack, carotid stenosis ≥ 50%, peripheral artery disease presumed to be of atherosclerotic origin (symptomatic or abnormal ankle-brachial index detected on screening), or aortic atherosclerosis.

### Checklist Components

Developed by authors of this review, the checklist includes core health behaviors (smoking, physical activity, diet, weight) and risk factors (blood pressure, cholesterol, diabetes). The checklist is based on the 2011 AHA and American College of Cardiology Foundation secondary prevention guidelines update,^27^ the AHA My Life Check – Life’s Simple 7,^28^ and the updated guidelines and expert consensus decision pathways from the AHA, American College of Cardiology, and the European Society of Cardiology/European Atherosclerosis Society for the management of blood pressure,^29^ dyslipidemias,^20,30,31,32^ overweight and obesity,^33^ and diabetes.^34^

The checklist includes a header to assist in identifying patients with ASCVD followed by 13 high-yield items focused on pharmacological and nonpharmacological management based on the strength of recommendations and quality of evidence. A brief description of each checkpoint was included while avoiding excess information that may burden physicians and contribute to inaction and therapeutic inertia. The checklist is not meant to be inclusive of all ASCVD secondary prevention management tactics but, rather, serve as an overall reminder of general indications, and physicians are encouraged to refer to the relevant guidelines for additional details. Therefore, specific medication names, dosages, titrations, and adverse events were purposely avoided in the checklist but will be made available in the checklist supporting tools. However, for certain novel cardiometabolic medications, including sodium-glucose cotransporter 2 (SGLT2) inhibitors and glucagon-like peptide-1 receptor agonists (GLP-1RA), more clinical practice and safety profile descriptions were added since many providers may be unfamiliar with these agents, leading to suboptimal implementation of the recommendations.

**Figure 1:**
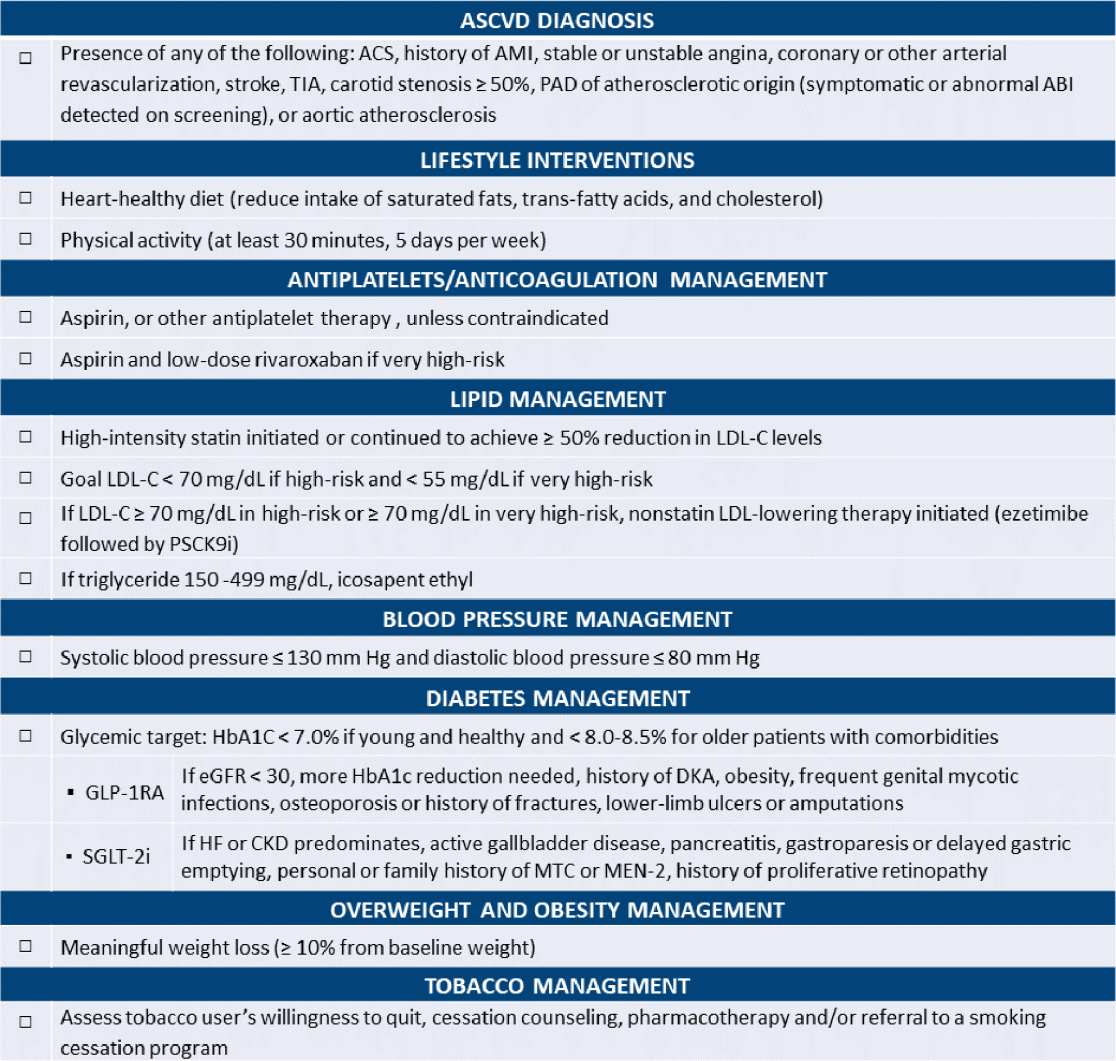
Checklist for secondary prevention of established atherosclerotic cardiovascular disease (ASCVD). ACS: acute coronary syndrome; AMI: acute myocardial infarction; TIA: transient ischemic attack; PAD: peripheral artery disease; ABI: ankle brachial index; LDL-C: low-density lipoprotein cholesterol; PSCK9i: proprotein convertase subtilisin/kexin type 9 inhibitor; HbA1c: glycated hemoglobin; GLP-1RA: glucagon-like peptide-1 receptor agonist; eGFR: estimated glomerular filtration rate; DKA: diabetic ketoacidosis; SGLT-2i: sodium-glucose cotransporter-2 inhibitor; HF: heart failure; CKD: chronic kidney disease; MTC: medullary thyroid cancer; MEN-2: multiple endocrine neoplasm type 2.

The checklist was reviewed by intended users and relevant experts including a panel of cardiology and primary care physicians at Houston Methodist Hospital, and written critiques of the checklist were provided. All reviews and feedback on the merit and utility of the checklist were addressed in the final version (***Figure 1***).

## Checklist Supporting Tools and Dissemination

The ASCVD secondary prevention checklist is one component of a decision aid designed to avoid omission of effective therapies, reduce treatment delays, and guide optimal care for patients with established ASCVD. In parallel with the checklist, supporting tools can improve workflow, reduce barriers, and facilitate the ultimate goal of implementing the best evidence-based clinical practice for patients with established ASCVD.

Physicians at Houston Methodist Hospital are currently developing comprehensive patient education materials explaining the evidence behind the ASCVD secondary prevention guideline recommendations. Multiple channels for communicating educational messages can be used, including traditional in-person lectures, online videos, and educational handbooks. Each core component of the secondary prevention checklist can be further illustrated using various mediums with an emphasis on current best-available scientific evidence and guideline recommendations. Upon the checklist’s initial implementation, targeted providers would receive instructions on its use as well as background information regarding its development and the basis of the recommendations. Additionally, patient education materials would potentially increase awareness of the available pharmacological and nonpharmacological treatment strategies and goals of management.

**Figure 2:**
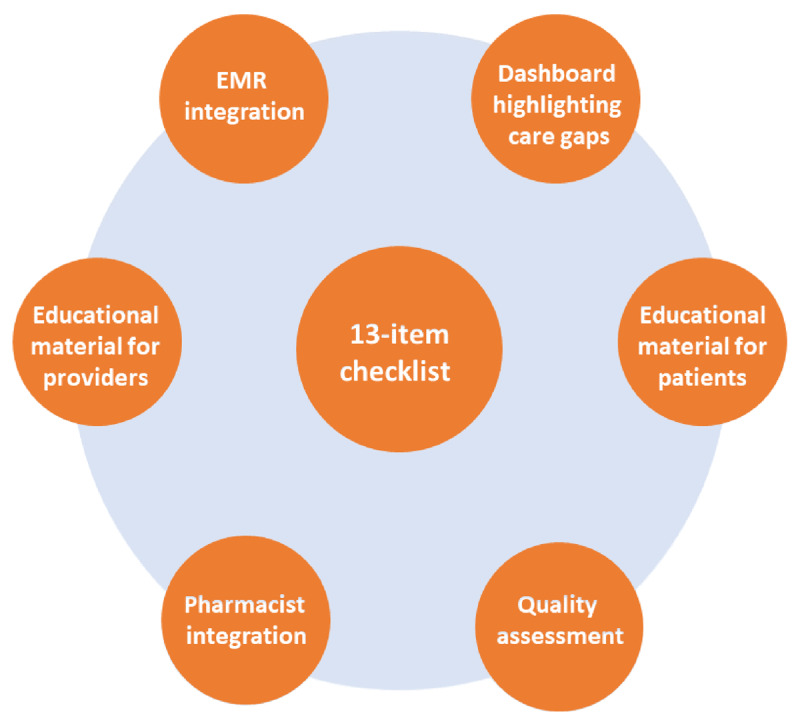
A proposed comprehensive tool for enhanced guideline-directed management in the secondary prevention of atherosclerotic cardiovascular disease. EMR: electronic medical record.

Using data from electronic medical records (EMRs), dashboards can be created to identify patients who are not receiving optimal preventive care based on defined algorithms. Physicians would be able to receive performance feedback with information regarding gaps in patient care. Additionally, pharmacists can be integrated into care delivery to help facilitate appropriate prescriptions with appropriate physician supervision. Finally, the checklist can be integrated into EMRs to facilitate timely feedback, identify gaps in care, and efficiently streamline prescription of effective therapies. The proposed comprehensive tool for enhanced guideline-directed management in the secondary prevention of ASCVD is illustrated in ***Figure 2***.

## Quality Improvement Measures

Quality improvement refers to the quality of the proposed comprehensive tool itself as well as the quality of care delivered to patients with ASCVD. Every application of the checklist and every invited review of the checklist and supporting tools provides information that may be useful in improving the checklist, and the checklist developers should review and address the rising issues at appropriate intervals. Furthermore, the proposed comprehensive tool should be updated as new guidelines are released.

Key indicators of quality can be monitored at the patient, provider, and system levels throughout the implementation of the checklist and supporting tools. EMR-based dashboards can be created and used to track treatment metrics specific to the selected patient population to assess potential improvement and persisting gaps in care—for example, tracking prescription of high-intensity statins and monitoring LDL-C levels and using this feedback to intensify lipid-lowering therapy when indicated. Other healthcare quality metrics that can be monitored include cardiovascular risk factor control, patient outcomes and satisfaction, and the overall impact of the checklist and associated tools on healthcare quality metrics. Furthermore, EMR integration can be of great value in providing performance feedback concerning specific points in the checklist. Understanding these quality measures can further aid in identifying certain gaps in the delivery of care where other intervention approaches may be needed. Future studies assessing the impact of the proposed ASCVD checklist and supporting tools on quality of care are needed and could use the Plan-Do-Study-Act framework for evaluation.

## Target of the Comprehensive Tool

The proposed comprehensive tool can be used by key stakeholders in the outpatient care of patients with established ASCVD, specifically primary care physicians and cardiologists. The checklist instructions can be delivered to these physicians to ensure their familiarity with the overall purpose and objectives and to receive feedback for improvements. Prior to its implementation, educational sessions can be provided to physicians caring for those with established ASCVD. Physicians will also be provided with snapshots of future supporting tools including educational materials for patients, dashboards, pharmacy resources, and EMR integration. After physicians are familiar with the tool and have received initial instructions, the checklist would be provided to physicians for use.

## Discussion

We propose a framework of a comprehensive tool for enhanced guideline-directed management in the secondary prevention of ASCVD. This aid consists of a 13-point checklist and supporting tools of what we consider to be essential components of effective and evidence-based pharmacological and nonpharmacological care for secondary prevention in patients with established ASCVD. This comprehensive tool can be used in an outpatient setting by primary care providers and cardiologists who provide direct medical care for patients with established ASCVD.

Guidelines are valuable resources that attempt to incorporate evidence from research into clinical practice, reduce inappropriate variability in practice, and provide the best available evidence to support clinical decision-making in order to improve quality of care, patient outcomes, and cost-effectiveness.^35^ An abundance of high-quality studies endorsed by current guidelines support the importance of secondary prevention in ASCVD. Despite this and the efforts from medical societies to disseminate knowledge and protocols, appropriate treatment remains an unaccomplished goal for a significant percentage of patients with established ASCVD. Reasons for such an evident care gap are likely multifactorial and may include socioeconomic determinants, noncompliance, and health literacy of the patient. However, clinicians may also play a role in the form of prescription inertia, lack of familiarity with or motivation to follow updated and often complex guidelines, time restrictions, and discomfort using novel therapeutics.^26^ If not collected in a simple user-friendly format, the abundance of data and recommendations can be daunting for specialists and primary care providers alike.

Targeting ASCVD with a guideline-based checklist has several advantages. First, there is tremendous potential to reduce the burden of recurrent ASCVD events given the major gaps in care for secondary prevention. Second, multiple preventive therapies are available for these patients, all with proven cardiovascular benefits and endorsement by guideline recommendations. Third, population-health–based approaches can be used to narrow gaps in preventive care due to the availability of EMRs, clear algorithms to identify ASCVD, and available treatments.

The potential benefits for patients are numerous. First, a focused checklist with supporting tools can help reduce gaps in care, which may have a meaningful impact on patient outcomes and quality of care. Second, more intensive education programs may help improve guideline implementation and adherence. Previous research indicates that physician attitude toward the ideal goals of ASCVD management is one of the main reasons for suboptimal treatment.^16^ Third, EMR integration of our proposed checklist can extend the use of EMRs to identify patients with ASCVD and gaps in care that need to be addressed and confirm that necessary management practices are followed. Similarly, EMRs can be used to identify patients at very high risk for ASCVD who require more intense treatment and monitoring. In addition, EMRs can be a great source of data collection that can be used to assess outcomes and quality of care and for continuous enhancement of the healthcare model. Finally, this tool can provide a framework for rapid-cycle implementation for other types of cardiovascular diseases—ie, heart failure—that have existing gaps in care.

Use of checklist-inspired tools has been identified as one of five critical strategies to enhance physicians’ use of clinical guidelines and help close the gap between knowledge and practice.^21^ Checklists and checklist-inspired tools have been pivotal in promoting safety and reducing errors outside of the medical field. Initially developed in aviation, these tools demonstrated their utility in guiding users through complex tasks with a step-by-step approach that reduces the likelihood of errors and aid in recalling critical elements and actions. Providing a quick, easily accessible reminder of appropriate actions and guiding the user through complex tasks, a checklist accomplishes its goal of facilitating appropriate care that is consistent and provided to the appropriate patient.

The positive impact of checklists on patient care has been documented in multiple fields. For example, implementation of a surgical safety checklist resulted in almost a 50% reduction in death rates and complications among patients undergoing noncardiac surgery.^36^ Likewise, the care bundles for preventing ventilator-acquired pneumonia in the intensive care setting^37^ and for sepsis management resulted in significant improvement in mortality outcomes in critically ill patients.^38^ On a smaller scale, other studies looked at locally developed inexpensive checklists or bundles to optimize patient safety. A study by Basoor and colleagues showed that checklist use in surgical rounds improved documentation of key aspects of patient care, whereas a study by Krishnamohan et al. showed that a heart failure management checklist was associated with better quality of care and decreased readmission rates for patients admitted with heart failure.^39,40^ The application of a checklist and its impact on clinical management is rarely a one-step process. It requires changes in the system as well as changes in provider behavior.

## Conclusion

We propose a framework for a comprehensive checklist tool that provides enhanced guideline-directed management in the secondary prevention of ASCVD. This approach can potentially have a meaningful impact on patient outcomes and quality of care and may help improve guideline implementation and adherence. Furthermore, it may help reduce the existing gaps in care between guidelines and clinical practice and can also be used for other types of cardiovascular diseases that have similar existing gaps in care.

## Key Points

- There are existing gaps in the management of patients with established atherosclerotic cardiovascular disease (ASCVD), some of which are related to barriers to guideline implementation.
- A framework for a comprehensive tool, including a checklist and supporting tools, is proposed for enhanced guideline-directed management in the secondary prevention of ASCVD.
- A 13-point checklist of pharmacological and nonpharmacological management based on clinical evidence and published guidelines was created. A brief description of each of the checkpoints was included while avoiding excess information that may burden physicians and contribute to inaction and therapeutic inertia.
- Implementation and use of a comprehensive checklist tool has the potential to reduce gaps in care, positively impact patient outcomes and quality of care, improve guideline implementation and adherence, and optimize the use of electronic medical records (EMRs) to identify patients with ASCVD and gaps in care that need to be addressed and/or those who are at risk of developing ASCVD.
- Checklist supporting tools consist of comprehensive educational materials that can be delivered to providers via in-person instruction, online videos, and educational handbooks and can be integrated into EMRs to identify gaps in care and quality.
